# Chloroform

**Published:** 1848-03

**Authors:** 


*Chloroform.*—In a paper published in the *Monthly Jour. of Med. Sci.*, for December, Prof. Simpson gives some further particulars in regard to chloroform, which we extract.

"*Physiological Effects.*—After the first two or three full inspirations, a feeling of warmth and excitation, radiating from the chest to the extremities; followed by whirring noises in the ears; a sensation of vibratory thrilling and benumbing throughout the body; with, betimes, rapid loss of sensation and motion, and at last of consciousness. Often before total unconsciousness supervenes, the patient, guided by instinct rather than volition and reason, makes an effort to get rid of the inhaling vapor and handkerchief, as if it interfered with free respiration. During the full anæsthetic sleep produced by chloroform, sometimes no mental action goes on, or at least is remembered; in many others, the mind is active as in dreams. The respiration is usually at first soporose; the pupil sometimes natural, in others slightly contracted, in others dilated; the pulse is usually quickened ten or twenty beats at first, but afterwards falls to its normal rate, and, if the vapor is exhibited very long in very powerful doses, it comes down more and more below the natural standard; muscles of voluntary motion in general relaxed; more rarely cataleptic; still more rarely clonically contracted, as happens also occasionally with ether. In small doses, given slowly, its effects are exhilerating, and exactly like those generally following the inhalation of nitrous oxide gas.

"*Uses in Surgery.*—1. To relax the muscles in reducing dislocations, &c.: 2. To avert the sufferings attendant on deep probing, and other painful but necessary modes of diagnostic examination and dressing; and 3, and principally, to annul the pain of operations by the caustic, ligature, or knife.

"*Uses in Midwifery.*—To diminish and annul the physical pains attendant on labor, and more especially those which accompany the passage of the child's head through the pelvic cavity and outlet, (the second stage of Denman.)

"*Uses in Medicine.*—1. As an *anti-spasmodic;* as in asthma, laryngismus, tetanus, and other spasmodic diseases, &c. I have used successfully the inhalation of ether to arrest the paroxysms of hooping-cough, dysmenorrhea, colic, and the pains attendant on the passage of biliary calculi. In a case of the most severe, at the same time painful, spasmodic twisting and convulsions of the extremities attending a second attack of chorea, I allowed the patient ether inhalation; and sometimes she lay under its influence for hours, with relief while its action lasted, but generally without sleep. Latterly the chloroform has both relieved the spasms and their attendant pain and procured sleep.—2. As an *anodyne* or *narcotic.* In neuralgia, I have seen chloroform stop the fit at once; in two other cases the pain remained absent only while the chloroform acted. A patient suffering under severe delirium tremens had remained awake for about seventy hours; a half ounce of laudunum, given at a single dose, failed to produce rest; ten hours afterwards, the inhalation of chloroform was immediately followed by several hours of critical sleep. What cases of insanity would it benefit? I have exhibited it in full doses in some cases of dementia, combined with excitement and wakefulness. They were all asleep in about a minute—and remained so for some time. In nothing does chloroform differ from ether more than in its soporific effects—when given in full doses and continued for sometime. 3. In small doses as a *diffusable stimulant;* to arrest the first commencement of ague, ephemera, &c.; in hysteria, &c. Perhaps it may be used by inhalation in small quantities when the stomach will not bear wine or other stimulants; in severe vomiting, fevers, &c. I have seen its inhalation at once dispel a sick headache.

"*Cautions.*—The liquid used should be sufficiently strong. Its proper sp. gr. is (as I have said) 1·480. It is certainly far too powerful an agent to be intrusted to nurses or unprofessional individuals. I have given it, up to this date, to above eighty persons, without the slightest bad result of any kind whatever in any one of them. The power, however, which we have with it, of bringing down the pulse, &c., shows that, if exhibited in too strong a dose, and given *uninterruptedly* for too great a length of time, it would doubtless produce serious consequences and even death. But, certainly, all its *full* anæsthetic and other influences may be perfectly obtained without allowing it to produce such depression as would be in any degree dangerous. Like many other agents, it may be powerful for evil as well as for good. I believe its great potency will be one great safeguard against its abuse.

"Its influence upon the blood, &c., the counter indications to its use, &c. &c., remain still to be ascertained."

*American Jour. Med. Science.*

